# Carotid bifurcation reconstruction as a novel CEA modification to address complex carotid lesions

**DOI:** 10.3389/fcvm.2026.1860792

**Published:** 2026-07-29

**Authors:** Zhaopeng He, Zhenduo Li, Kunyu Wang, Lei Zhang

**Affiliations:** Department of Vascular Surgery, the First Hospital of Hebei Medical University, Shijiazhuang, Hebei Province, China

**Keywords:** carotid atherosclerosis, carotid bifurcation reconstruction, carotid endarterectomy, complex carotid lesions, modified technique

## Abstract

**Objective:**

To evaluate the feasibility and short-term outcomes of carotid bifurcation reconstruction, a modified carotid endarterectomy (CEA) technique for complex carotid lesions, by comparing it with conventional CEA with patch angioplasty.

**Methods:**

A retrospective cohort study was conducted on patients who underwent carotid revascularization at our institution between September 2023 and November 2025. Patients receiving carotid bifurcation reconstruction surgery were assigned to the observation group (*n* = 21), and those receiving conventional CEA with patch angioplasty during the same period were assigned to the control group (*n* = 33). All procedures were performed by a single senior vascular surgeon. Baseline characteristics, preoperative imaging findings, intraoperative parameters, postoperative complications and follow-up data were collected and compared between the two groups.

**Results:**

A total of 54 patients were enrolled, with no significant differences in baseline demographic and clinical characteristics between the two groups (all *P* > 0.05). The median follow-up duration was 14 months (IQR 6–21 months) in the observation group and 16 months (IQR 8–23 months) in the control group, with no significant between-group difference (*P* = 0.4025) and no patient lost to follow-up. For the overall cohort, no significant between-group differences were found in total operative time (*P* = 0.106) and intraoperative shunt use rate (*P* = 0.083), while the observation group had significantly less intraoperative blood loss [100 (50, 100) mL vs. 120 (100, 155) mL, *P* = 0.008]. In patients without intraoperative shunt placement, the carotid cross-clamp time was significantly shorter in the observation group than in the control group (22.4 ± 4.1 min vs. 25.5 ± 3.4 min, *P* = 0.032). The observation group had a shorter postoperative hospital stay [5.0 (5.0, 6.0) d vs. 6.0 (5.0, 8.0) d, *P* = 0.005]. No major perioperative complications occurred in either group, and all observed adverse events were transient and resolved before discharge. During follow-up, no ipsilateral ischemic stroke or significant carotid restenosis (≥ 50%) was detected in either group.

**Conclusion:**

Carotid bifurcation reconstruction may be a feasible modified CEA technique for selected patients with complex carotid lesions.

## Introduction

Carotid artery stenosis (CAS) is primarily caused by atherosclerosis. Atherosclerotic plaques not only reduce cerebral blood flow due to luminal narrowing but also increase the risk of stroke through plaque rupture, thrombosis, or embolization to intracranial arteries. As a major modifiable risk factor for ischemic stroke, CAS accounts for 8%–15% of all ischemic cerebrovascular events worldwide (1). According to current clinical practice guidelines, treatment strategies for CAS include best medical therapy (BMT), carotid endarterectomy (CEA), and carotid artery stenting (2). Medical therapy remains the cornerstone of management, with a focus on risk factor control (e.g., hypertension, diabetes, dyslipidemia, smoking) and the use of antiplatelet therapy and statin therapy.

Recent large-scale trials have reshaped the treatment paradigm for asymptomatic and low-risk symptomatic carotid stenosis. The CREST-2 trial (3) demonstrated that BMT plus carotid artery stenting reduced stroke risk in patients with high-grade asymptomatic carotid stenosis, whereas CEA plus BMT did not show a significant advantage over medical therapy alone. The 2-year interim analysis of the ECST-2 trial (4) found no additional benefit of revascularization over BMT in asymptomatic or low-to-intermediate risk symptomatic patients. Nonetheless, for patients with complex lesions, high-risk plaque features, or progressive symptoms despite BMT, surgical revascularization remains a critical therapeutic option.

Previous studies have demonstrated that, compared with Western populations, East Asian patients represented by Chinese cohorts exhibit unique unfavorable anatomy, including higher carotid bifurcation, greater tortuosity, and more tandem lesions, which increase surgical difficulty and risk of CEA, making CEA-ineligible anatomy more prevalent (5).

To address more complex anatomical factors and lesion severity, our center has explored modified CEA techniques to overcome the limitations of existing procedures. This study introduces a novel CEA technique—carotid bifurcation reconstruction surgery—and conducts a retrospective comparative analysis with conventional CEA to evaluate its feasibility, safety and short-term clinical efficacy in the treatment of complex carotid lesions.

## Surgical technique

All procedures were performed under general anesthesia, and all patients underwent intraoperative angiography. The decision to perform CEA was definitively made preoperatively based on carotid duplex ultrasound and computed tomographic angiography (CTA) findings. Intraoperative angiography was not used to select between stenting and open surgery. Shunt placement was used selectively based on stump pressure, the degree of contralateral carotid stenosis, the integrity of the circle of Willis, and intraoperative neuromonitoring findings.

We begin by puncturing the right femoral artery and inserting a 5F vascular sheath, after which we perform aortography of the aortic arch using a pigtail catheter and guidewire. This allows us to assess the intracranial circulation. By acting as a preoperative reference for subsequent completion angiography, it allows for immediate assessment of cerebral blood flow improvement and early recognition of procedural complications such as residual stenosis or intimal dissection. Next, we make an oblique incision along the anterior border of the sternocleidomastoid muscle and fully dissect the common carotid artery (CCA), internal carotid artery (ICA), and external carotid artery (ECA), encircling each vessel with vascular tapes while taking care to preserve adjacent neurovascular structures.

After systemic heparinization, we apply vascular clamps to the ICA, ECA, CCA, and superior thyroid artery. The two groups adopt different arteriotomy and vessel closure strategies, as described separately below.

### Carotid bifurcation reconstruction (observation group)

For patients in the observation group, the arteriotomy is designed for bifurcation reconstruction. A longitudinal arteriotomy is made on the anterior wall of the CCA and extended cephalad to the apex of the carotid bifurcation, where the incision continues along the midline of the opposing walls of the ICA and ECA, with equal incision lengths in both arteries. The total length of the arteriotomy is individually adjusted according to the proximal and distal extent of the plaque.

The atherosclerotic plaque is carefully dissected, and the lumen is thoroughly irrigated with heparinized saline. We trim the vascular edges to align the internal and external incisions and initiate continuous suturing with 6-0 Prolene from the low point of the posterior wall (bifurcation base), extending it upward to the new apex. The anterior wall is then sutured from the low point upward to complete the closure.

### Conventional CEA with patch angioplasty (control group)

For patients in the control group, a standard longitudinal arteriotomy is made on the anterior wall of the CCA and extended cephalad along the anterior wall of the ICA, terminating 2–3 mm beyond the distal margin of the plaque without involving the ECA trunk. After careful dissection of the atherosclerotic plaque and thorough irrigation of the lumen with heparinized saline, patch angioplasty is performed to avoid luminal stenosis caused by primary closure. A suitably sized patch (expanded polytetrafluoroethylene [ePTFE] synthetic patch) is trimmed to a fusiform shape matching the length and width of the arteriotomy. A double-armed 6-0 Prolene suture is used for anastomosis: the suture is anchored at the distal apex of the ICA incision with the patch tip aligned, and each needle is passed proximally along one lateral margin of the arteriotomy in a continuous running fashion. The two sutures converge at the proximal apex of the arteriotomy at the CCA level, where they are tied securely to achieve adequate luminal augmentation and watertight closure.

**Figure 1:**
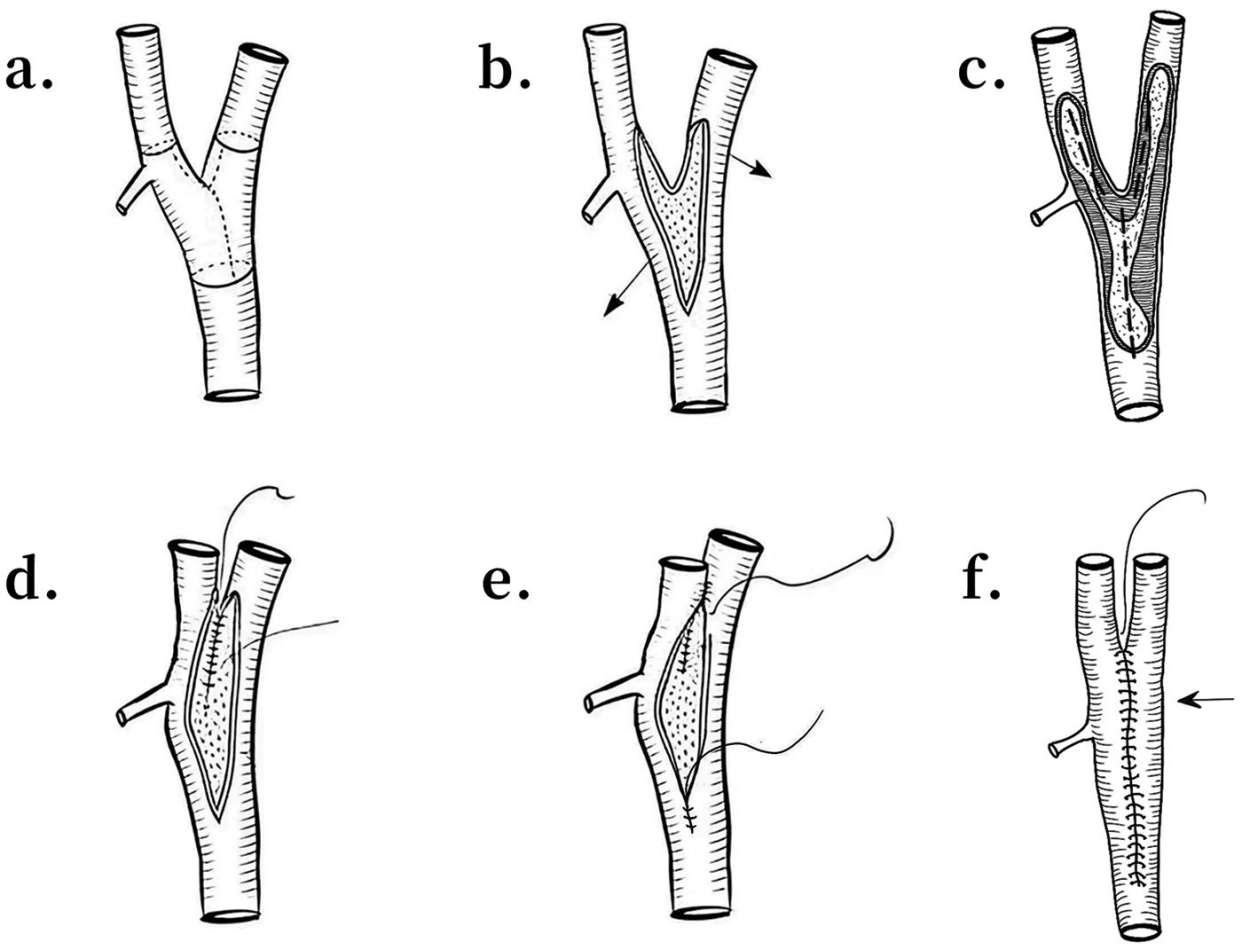
Schematic diagram of the operation. **(a)** Location of the carotid incision. **(b,c)** Carotid arteriotomy revealing the intima and plaque. **(d,e,f)** Suturing of the incision and completion of carotid bifurcation elevation.

**Figure 2:**
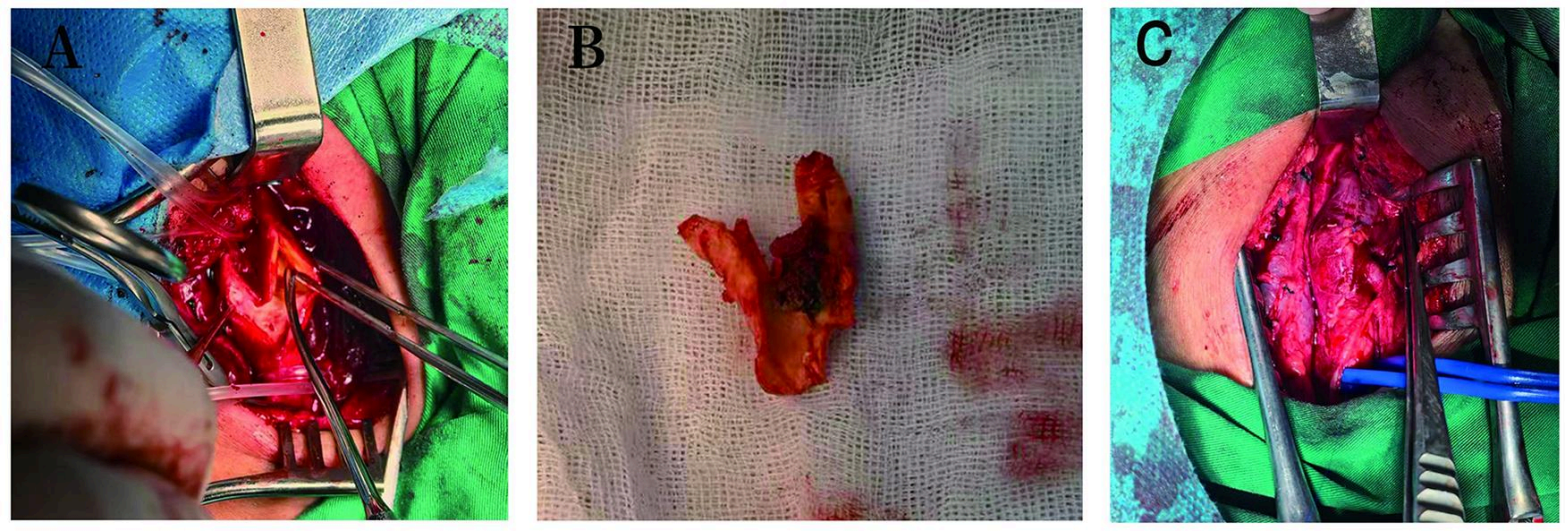
Intraoperative Images. **(A)** The carotid artery was incised. **(B)** Plaque dissection. **(C)** Completion of suturing.

**Figure 3:**
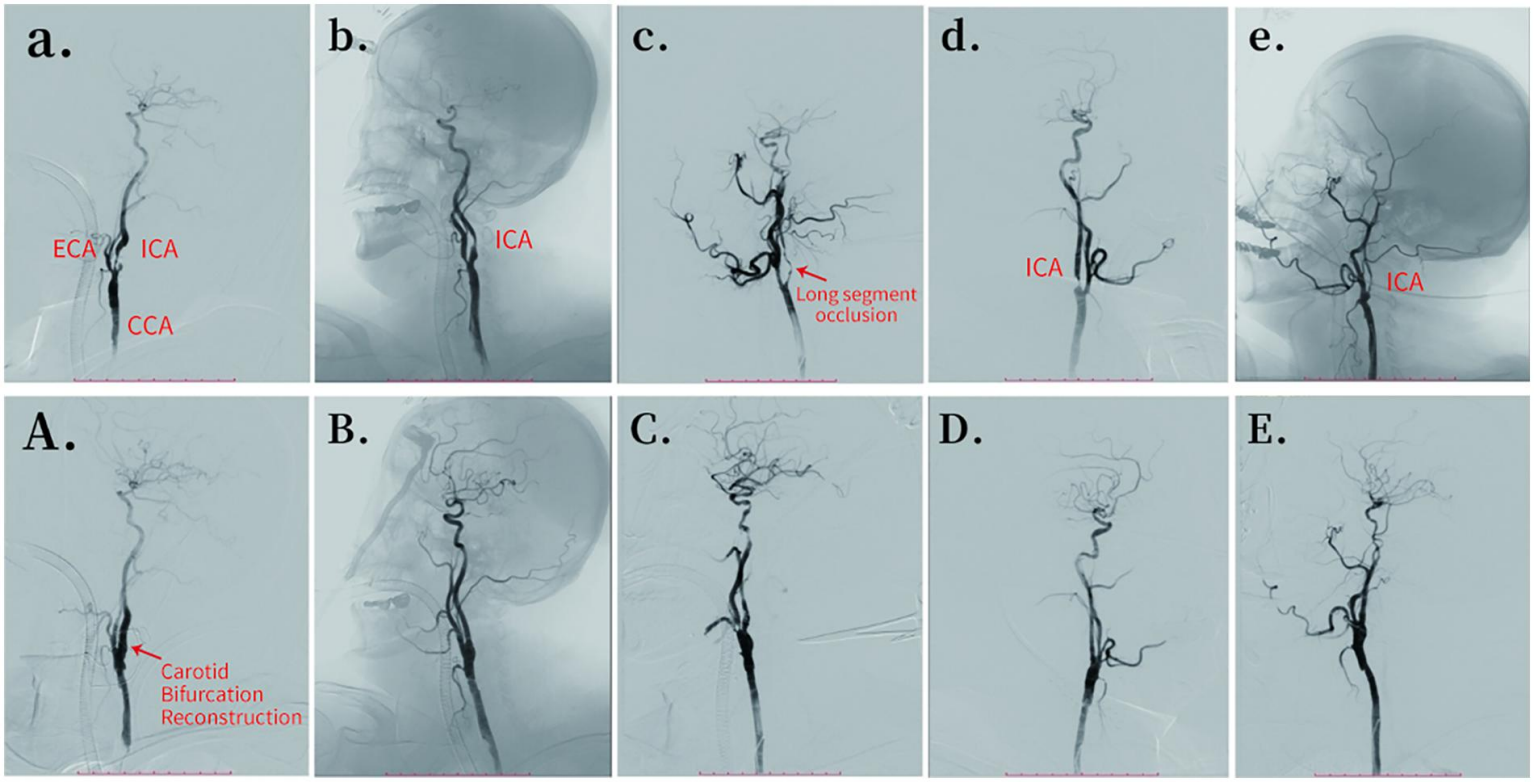
Preoperative and postoperative angiographic images of representative cases. Each lowercase label **(A–E)** corresponds to a distinct patient, showing the preoperative angiogram; the matching uppercase label **(A–E)** shows the postoperative angiogram of the same patient.

Following suture completion, we unclamp the ICA and immediately re-clamp it to evacuate air and debris, then sequentially release the ECA, CCA, and ICA clamps to restore flow. Finally, we perform completion angiography to confirm luminal patency. We use intraoperative stent implantation only as a bailout procedure for significant residual stenosis, intimal flap, or flow-limiting dissection, rather than as a planned alternative treatment strategy. After achieving hemostasis, we close the incision in layers. Figure 1 illustrates the key surgical procedure. Intraoperative photographs and preoperative–postoperative angiographic comparisons are presented in Figures 2, 3, respectively.

## Methods

This single-center retrospective cohort study consecutively enrolled patients who underwent carotid endarterectomy at our institution between September 2023 and November 2025. Patients who received carotid bifurcation reconstruction surgery were defined as the observation group, and patients who received conventional standard CEA with patch angioplasty during the same period were defined as the control group. All procedures were performed by a single senior vascular surgeon. Data were extracted from electronic medical records, the picture archiving and communication system (PACS), and outpatient follow-up records. Telephone interviews were conducted to supplement follow-up data.

All patients received guideline-recommended optimal medical therapy preoperatively. Surgical indications followed the 2023 European Society for Vascular Surgery (ESVS) guidelines (2): symptomatic patients with ≥50% stenosis within the preceding 6 months, and asymptomatic patients with severe stenosis (60%–99%) who were at high risk of stroke and have a reasonable life expectancy. Stenosis severity was measured using the North American Symptomatic Carotid Endarterectomy Trial (NASCET) criteria.

For all enrolled patients, both CEA and carotid artery stenting were formally evaluated as potential revascularization strategies during preoperative discussion, in accordance with the 2023 ESVS clinical practice guidelines. The treatment decision was made by comprehensively integrating lesion anatomy, plaque characteristics, patient comorbidities. The main rationales for prioritizing CEA over carotid artery stenting included:

Unfavorable anatomical factors for carotid artery stenting: long-segment (≥2 cm) heavily calcified plaques, which pose a high risk of stent malapposition and in-stent restenosis; type III aortic arch or anomalous supra-aortic branching that increase the difficulty of endovascular access.

Plaque-related high-risk features: ulcerated plaques or intraluminal thrombus, which are associated with an elevated risk of embolic stroke during carotid artery stenting procedures.

Patient-related factors: history of chronic kidney disease, to minimize contrast agent load and reduce the risk of contrast-induced nephropathy.

**Figure 4:**
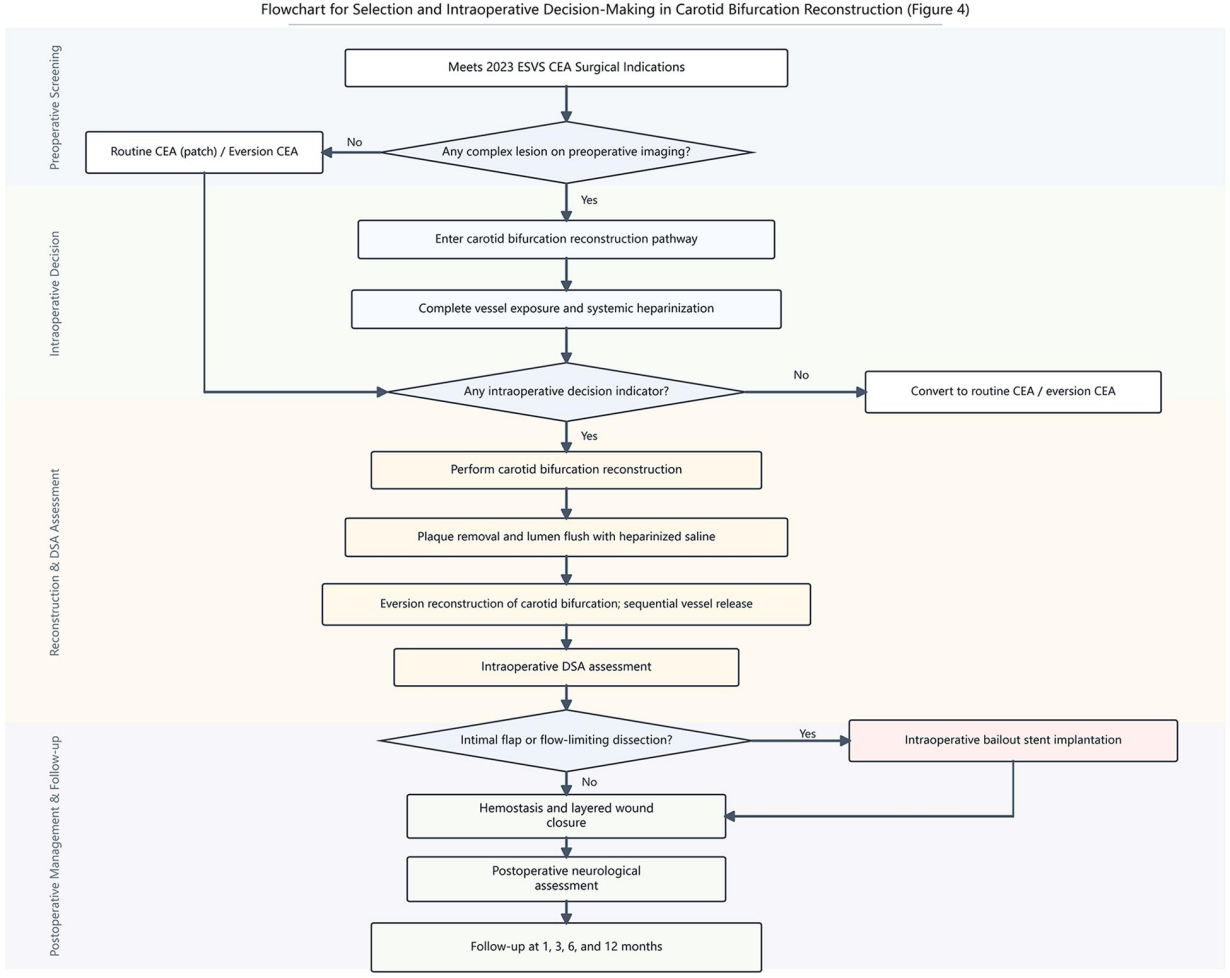
Flowchart of patient selection and intraoperative decision-making for carotid bifurcation reconstruction. This diagram illustrates the stepwise surgical decision pathway from preoperative indication screening, intraoperative lesion stratification, to intraoperative DSA evaluation and standardized postoperative follow-up. CEA, carotid endarterectomy; DSA, digital subtraction angiography; ESVS, European Society for Vascular Surgery.

Patients in the observation group represented a complex subgroup for whom conventional CEA with patch or eversion CEA was deemed technically challenging. The stepwise surgical decision algorithm and procedural flowchart are illustrated in Figure 4. The criteria for defining complex carotid lesions and selecting carotid bifurcation reconstruction included: (i) Long-segment atherosclerotic plaque (≥ 2 cm in length) at the origin of the ICA, concurrent with severe stenosis (≥ 70%) at the origin of the ECA; (ii) High carotid bifurcation located above the inferior border of the C3–C4 intervertebral disc on preoperative CTA; (iii) Severely calcified carotid plaque with circumferential calcification involving over 270° of the vessel circumference; (iv) Luminal diameter (≤ 3 mm) of the ICA distal to the plaque margin.

Any lesion meeting at least one of the above criteria was defined as a complex carotid lesion.

## Data collection included three categories

Baseline and Preoperative Data: Age, sex, symptoms, preoperative neurological status (modified Rankin Scale [mRS]), and imaging findings (carotid duplex ultrasound, CTA).

Intraoperative Data: Anesthesia type, total operative time, carotid cross-clamp time, shunt use, and technical details.

Postoperative and Follow-up Data: In-hospital complications (stroke, myocardial infarction, cranial nerve injury, hematoma, blood pressure abnormalities), length of stay, neurological recovery, and carotid patency and restenosis.

Postoperative follow-up was conducted at 1 month, 3 months, 6 months, 12 months after surgery, and annually thereafter. Follow-up visits included clinical neurological assessment (mRS) and vascular imaging evaluation. Carotid duplex ultrasound (CDUS) was performed as the first-line imaging modality at all scheduled follow-up time points. CTA was performed for patients with suspicious findings on CDUS (e.g., elevated flow velocity, unclear lumen morphology) to confirm the presence and severity of restenosis. Restenosis was defined as luminal stenosis ≥50% measured by the NASCET criteria.

The primary endpoint of this study was the occurrence of ipsilateral carotid restenosis or ipsilateral stroke during follow-up. Secondary endpoints included cranial nerve injury, myocardial infarction, and neurological functional outcome. For patients without endpoint events, follow-up was censored at the uniform study cutoff date (January 1, 2026). Overall follow-up time was defined as the interval from the date of surgery to either the date of endpoint event occurrence or the study cutoff date. The median follow-up time and interquartile range (IQR) for the entire cohort are reported.

Statistical analysis was performed using R software (version 4.4.2). Continuous variables were tested for normality using the Shapiro–Wilk test. Normally distributed data are presented as mean ± standard deviation and compared using the independent samples t-test. Non-normally distributed data are presented as median (interquartile range, IQR) and compared using the Mann–Whitney U test (Wilcoxon rank-sum test). Categorical variables are presented as frequency (percentage) and compared using the *χ*^2^ test or Fisher's exact test where appropriate. A two-sided *P* value < 0.05 was considered statistically significant.

The study was approved by the Medical Ethics Committee of our hospital. Identifiable patient private information was not used for this study; all data included in the analysis were de-identified and anonymized to ensure patient privacy. Informed consent was waived due to the use of de-identified retrospective data. Written informed consent for publication of the images was obtained from the patients. The study was conducted in accordance with the Declaration of Helsinki.

## Results

A total of 54 patients were finally enrolled, including 21 in the observation group and 33 in the control group. The baseline demographic and clinical characteristics of the two groups are summarized in Table 1. There were no significant differences between the two groups in age, sex, body mass index, smoking status, alcohol drinking status, or comorbidities including hypertension, diabetes mellitus, hyperlipidemia, coronary artery disease, cerebrovascular disease and heart failure (all *P* > 0.05), indicating good baseline comparability between the two groups.

**Table 1:** Baseline demographic and clinical characteristics of the two groups.

Characteristics	Control group (*n* = 33)	Observation group (*n* = 21)	Statistic	*P* value
Demographics				
Age, years	63.24 ± 7.45	64.48 ± 5.79	t = 0.681	0.4988
Sex, male	18 (54.5)	10 (47.6)	*χ*^2^= 0.047	0.8280
BMI	23.70 ± 2.92	24.99 ± 3.15	t = 1.496	0.1424
Lifestyle				
Smoking, current	14 (42.4)	11 (52.4)	χ^2^ = 0.190	0.6633
Alcohol use, current	15 (45.5)	8 (38.1)	χ^2^ = 0.060	0.8019
Comorbidities				
Hypertension	18 (54.5)	16 (76.2)	χ^2^ = 1.734	0.1879
Diabetes mellitus	9 (27.3)	8 (38.1)	χ^2^= 0.285	0.5932
Hyperlipidemia	15 (45.5)	12 (57.1)	χ^2^ = 0.312	0.5766
CAD	4 (12.1)	4 (19.0)	Fisher's exact test	0.6966
Cerebrovascular disease	11 (33.3)	12 (57.1)	χ^2^ = 2.081	0.1491
Heart failure	1 (3.0)	0 (0.0)	Fisher's exact test	1.0000

Continuous variables were tested for normality prior to comparison. Normally distributed data are presented as mean ± standard deviation and compared using the independent samples t-test. Categorical variables are presented as *n* (%) and compared using the χ^2^ test or Fisher's exact test where appropriate.

BMI, body mass index; CAD, coronary artery disease.

The preoperative clinical symptoms, neurological function and imaging characteristics of the two groups are shown in Table 2. There were no significant differences between the two groups in symptomatic status, preoperative mRS score, lesion laterality and stenosis severity (all *P* > 0.05). However, compared with the control group, the observation group had a significantly higher proportion of mixed-site plaques (57.1% vs. 15.2%, *P* = 0.0033) and long-segment plaques ≥ 2 cm (66.7% vs. 12.1%, *P* < 0.001), suggesting more complex lesion anatomy in the observation group.

**Table 2:** Comparison of preoperative clinical and imaging characteristics between the two groups.

Characteristics	Control group (*n* = 33)	Observation group (*n* = 21)	Statistic	*P* value
Symptomatic status				
Asymptomatic Dizziness (non-specific) Ischemic stroke TIA	14 (42.4) 10 (30.3) 6 (18.2) 3 (9.1)	5 (23.8) 7 (33.3) 5 (23.8) 4 (19.0)	Fisher's exact test	0.4801
Preoperative functional status (mRS)				
0 1 2	28 (84.8) 5 (15.2) 0 (0.0)	16 (76.2) 4 (19.0) 1 (4.8)	W = 314	0.3997
Lesion laterality				
Left Right Bilateral significant stenosis	14 (42.4) 13 (39.4) 6 (18.2)	8 (38.1) 6 (28.6) 7 (33.3)	χ^2^ = 1.71	0.4253
Stenosis severity (NASCET Criteria)				
Severe (70%–99%) Moderate (50%–69%) Mild (<50%)	30 (90.9) 3 (9.1) 0 (0.0)	20 (95.2) 1 (4.8) 0 (0.0)	W = 331.5	0.5707
Plaque location (Primary site)				
Isolated internal carotid artery Mixed-site plaque	28 (84.8) 5 (15.2)	9 (42.9) 12 (57.1)	χ^2^ = 8.6341	0.0033
Plaque length				
≥ 2 cm < 2 cm	4 (12.1) 29 (87.9)	14 (66.7) 7 (33.3)	W = 157.5	*P* < 0.001

Categorical variables are presented as *n* (%).

mRS, modified Rankin Scale; NASCET, North American Symptomatic Carotid Endarterectomy Trial; TIA, transient ischemic attack.

All procedures were successfully completed in both groups. The intraoperative parameters are compared in Table 3.

**Table 3:** Comparison of surgical details between the two groups.

Intraoperative parameters	Control group (*n* = 33)	Observation group (*n* = 21)	Statistic	*P* value
Operative laterality, Left	18 (54.5)	11 (52.4)	χ^2^ = 0.00	1.000
Carotid cross-clamp time, min	25.5 ± 3.4	22.4 ± 4.1	t = 2.26	0.032
Total operative time, min	153 (130, 184)	139 (113, 160)	W = 438	0.106
Intraoperative shunt	17 (51.5)	5 (23.8)	χ^2^ = 3.01	0.083
Surgical drain placement	33 (100.0)	21 (100.0)	—	—
Heparin use (5,000 IU)	33 (100.0)	21 (100.0)	—	—
Protamine use	0 (0.0)	0 (0.0)	—	—
Fibrin sealant use	16 (48.5)	5 (23.8)	χ^2^ = 2.33	0.127
Intraoperative blood loss, mL	120 (100, 155)	100 (50, 100)	W = 432.5	0.008

Carotid cross-clamp time was measured only in patients without intraoperative shunt placement (17 cases in the control group, 5 cases in the observation group). “—” indicates no statistical test was performed.

There was no significant difference in operative laterality between the two groups (*P* = 1.000). The total operative time was comparable between the observation group and the control group [139 (113, 160) min vs. 153 (130, 184) min], with no statistically significant difference (W = 438, *P* = 0.106). The rate of intraoperative shunt placement did not differ significantly between the two groups (23.8% vs. 51.5%, *χ*^2^ = 3.01, *P* = 0.083). In contrast, intraoperative blood loss was significantly lower in the observation group than in the control group [100 (50, 100) mL vs. 120 (100, 155) mL; W = 432.5, *P* = 0.008].

For the subgroup of patients who did not receive intraoperative shunt placement (17 cases in the control group and 5 cases in the observation group), the carotid cross-clamp time was significantly shorter in the observation group than in the control group (22.4 ± 4.1 min vs. 25.5 ± 3.4 min; t = 2.26, *P* = 0.032).

Regarding perioperative medication and device use, surgical drain placement and intraoperative heparinization (5000 IU) were universally applied in both groups, and no protamine was administered in any patient. The use rate of fibrin sealant showed no significant between-group difference (23.8% vs. 48.5%, *χ*^2^ = 2.33, *P* = 0.127).

One patient in the observation group underwent intraoperative stent implantation as a bailout procedure for residual distal stenosis detected on completion angiography, and subsequent angiography confirmed satisfactory luminal patency.

The postoperative length of hospital stay and complications are compared in Table 4. The postoperative hospital stay in the observation group was significantly shorter than that in the control group [5.0 (5.0, 6.0) d vs. 6.0 (5.0, 8.0) d, *P* = 0.005].

**Table 4:** Comparison of postoperative length of hospital stay and major postoperative complications between the two groups.

Characteristics	Control group (*n* = 33)	Observation group (*n* = 21)	Statistic	*P* value
Postoperative length of hospital stay, days	6.0 (5.0, 8.0)	5.0 (5.0, 6.0)	W = 498.5	0.005
Any postoperative complications	2 (6.1)	3 (14.3)	Fisher's exact test	0.366
Intimal flap dislocation	0 (0.0)	1 (4.8)	—	—
Neck hematoma	1 (3.0)	0 (0.0)	—	—
Surgical site infection	0 (0.0)	0 (0.0)	—	—
Recurrent laryngeal nerve injury (hoarseness)	1 (3.0)	1 (4.8)	—	—
Hypoglossal nerve palsy	0 (0.0)	1 (4.8)	—	—
TIA	0 (0.0)	0 (0.0)	—	—
Ischemic stroke	0 (0.0)	0 (0.0)	—	—
Carotid restenosis	0 (0.0)	0 (0.0)	—	—

Categorical variables are presented as *n* (%). “—” indicates no statistical test was performed due to extremely low event counts.

TIA, transient ischemic attack.

The overall incidence of postoperative complications showed no statistically significant difference between the two groups (14.3% vs. 6.1%, *P* = 0.366). In terms of specific complications, one case of intimal flap dislocation, one case of recurrent laryngeal nerve injury (hoarseness) and one case of hypoglossal nerve palsy occurred in the observation group; one case of neck hematoma and one case of recurrent laryngeal nerve injury occurred in the control group. No surgical site infection, TIA, ischemic stroke or carotid restenosis occurred during hospitalization in either group. All complications were transient and resolved with conservative management before discharge.

The median follow-up duration was comparable between the two groups (observation group: 14 months, IQR 6–21 months; control group: 16 months, IQR 8–23 months; *P* = 0.4025), with variation in individual follow-up length attributed to staggered surgical dates across the entire cohort. No patient was lost to follow-up in either group, and no ipsilateral ischemic stroke or significant carotid restenosis (≥ 50%) was detected during the entire follow-up period.

## Discussion

Preliminary findings from this single-center retrospective cohort study compared carotid bifurcation reconstruction with conventional CEA with patch angioplasty in patients with carotid atherosclerotic stenosis. Despite more complex lesion anatomy in the observation group, the modified technique appeared to be associated with shorter carotid cross-clamp time, reduced intraoperative blood loss, and faster postoperative recovery, while maintaining comparable perioperative safety in the present cohort. These preliminary results suggest that carotid bifurcation reconstruction may hold potential clinical utility in the management of challenging carotid lesions.

In the context of evolving evidence, the role of carotid artery stenting is being continuously refined (3, 4). Our cohort focused on patients with complex anatomical lesions who are often excluded from large randomized trials, and for whom carotid artery stenting may be technically challenging. For this specific subgroup, CEA remains an established treatment for appropriately selected patients with carotid stenosis, although the relative roles of CEA, carotid artery stenting, and BMT continue to evolve, particularly in asymptomatic disease (6–8) Technical refinements in CEA have focused on reducing complications, expanding applicability to complex lesions, and improving long-term patency (9, 10).

Conventional CEA with primary closure is straightforward but associated with restenosis rates of 13.6–27.0% and long-term stroke rates of 3.2%–4.6%. Patch angioplasty substantially reduces the risk of postoperative stroke and restenosis, but is associated with potential complications including infection, pseudoaneurysm, and arterial thrombosis (11–13) Moreover, patch angioplasty prolongs operative and cross-clamp times, increasing cerebral ischemia risk and often necessitating shunt placement (9).

Eversion CEA eliminates the need for patch material, offers shorter operative times, and yields lower restenosis rates (12). However, it is less suitable for long-segment or high-positioned plaques and is associated with postoperative hypertension in up to 53.8% of cases as a result of carotid sinus nerve transection (14). Modified eversion techniques have further reduced operative times and nerve injury but remain limited in their applicability to complex lesions (15, 16).

To address the technical limitations of conventional and eversion CEA, investigators worldwide have proposed numerous technical modifications. Kotsis et al. shifted the incision away from the carotid sinus to mitigate postoperative hypertension (17). Shukuzawa Kota et al. adopted a sequence of clamping the CCA and ECA prior to ICA dissection for eversion CEA, which lowered the risk of intraoperative embolic stroke, yet remained challenging for high-risk and complex carotid plaques (18). Some scholars have also developed optimized approaches for the carotid bifurcation. Nett et al. reported a 10-year neurological death-free survival rate of 92.5% in 181 modified CEA cases, but the technique was technically demanding and has not been widely adopted owing to concerns about vascular tortuosity and luminal irregularity (19). We have modified the carotid arteriotomy design of the procedure described by Nett et al., which employs an anterior carotid wall arteriotomy. Furthermore, we contend that this optimized arteriotomy strategy exhibits distinct clinical advantages in the management of complex carotid lesions.

Our technique extends the incision from the anterior CCA wall to the opposite walls of the ICA and ECA, leveraging the similar wall architecture to achieve luminal expansion. Intraoperative blood loss and postoperative incisional hematoma are intrinsically linked, both reflecting surgical trauma and anastomotic hemostatic efficacy. In our cohort, the observation group had significantly reduced intraoperative blood loss, which may be partially explained by the similar wall architecture of the internal and external carotid arteries. This structural similarity may support more precise and uniform tissue coaptation during suturing, potentially reducing anastomotic oozing. Consistently, no neck hematoma developed in the observation group, whereas one case occurred in the control group, which was likely associated with anastomotic oozing from patch suturing. This reduced bleeding-related risk also partially accounts for the faster postoperative recovery and shorter hospital stay in the observation group.

In the comparative analysis, even with more complex lesions, the observation group achieved a significantly shorter cross-clamp time than the control group, which is critical for reducing the risk of intraoperative cerebral ischemia, and may be attributed to the simplified suturing technique. Meanwhile, the shunt use rate in the observation group was 23.8%, lower than 51.5% in the control group, although the difference did not reach statistical significance, possibly due to the limited sample size. Reduced shunt dependence can avoid endothelial injury caused by shunt placement.

By enabling simultaneous plaque resection across the internal, external, and common carotid arteries, our modified technique restores anterograde flow in the external carotid artery and may improve extracranial-intracranial collateral circulation, which may confer a potential benefit in reducing stroke and restenosis risk (20). This putative benefit remains to be validated in larger cohorts.

For patients with high carotid bifurcations (21), this technique reduces suture difficulty and enhances clinical applicability. However, adequate exposure of the external carotid artery is necessary for anastomosis, while protecting the surrounding nerves and vessels.

Technical success was achieved in all patients in both groups. One patient in the observation group required intraoperative stenting for flow-limiting intimal flap. It is a common complication across all CEA techniques. The single case in our observation group was mainly attributed to the complexity of the lesion itself, rather than an inherent defect in the surgical design. No major perioperative complications occurred in either group, and no ipsilateral stroke or significant restenosis was observed during follow-up in the entire cohort, indicating acceptable short-term safety and patency of the modified technique.

This study has several limitations. First, it is a single-center retrospective study with a relatively small sample size, and the allocation of surgical techniques was not randomized, which may introduce selection bias. Second, the observation group had more complex lesions, which may affect the comparability of efficacy outcomes to some extent. Third, the follow-up duration is relatively short, and long-term outcomes beyond 3 years, especially long-term restenosis rate and stroke incidence, are not yet available. Future multicenter prospective randomized controlled trials with larger cohorts and extended follow-up are needed to further validate the safety and long-term efficacy of this technique, particularly in elderly patients and those with significant comorbidities.

## Conclusion

Compared with conventional CEA with patch angioplasty, carotid bifurcation reconstruction appears to be a feasible technical modification for selected patients with complex carotid lesions, with acceptable short-term safety in this cohort. In the present study, it obviates the need for patch angioplasty while maintaining adequate luminal diameter, and may confer potential benefits in shortening carotid cross-clamp time, reducing intraoperative blood loss, and accelerating early postoperative recovery. The short-term findings are encouraging but should be regarded as hypothesis-generating. Larger prospective comparative studies with longer follow-up are required to verify its long-term efficacy and clarify its appropriate clinical application scope.

## Data Availability

The raw data supporting the conclusions of this article will be made available by the authors, without undue reservation.
